# Young people’s perspectives of thyroid cancer screening and its harms after the nuclear accident in Fukushima Prefecture: a questionnaire survey indicating opt-out screening strategy of the thyroid examination as an ethical issue

**DOI:** 10.1186/s12885-022-09341-6

**Published:** 2022-03-03

**Authors:** Sanae Midorikawa, Akira Ohtsuru

**Affiliations:** 1 Faculty of Human Life Science, Miyagi Gakuin Women’s University, 9-1-1 Sakuragaoka, Aobaku, Sendai, 981-8557 Japan; 2 Department of First Internal Medicine, Nagasaki University, Nagasaki, Japan

**Keywords:** Overdiagnosis, Thyroid cancer, Decision-making, Screening, Nuclear accident

## Abstract

**Background:**

Overdiagnosis of thyroid cancer has become a major global medical issue. Ultrasound-based thyroid cancer screening has promoted overdiagnosis, and recently international recommendations state that it should not be conducted, even after a nuclear accident. The Fukushima thyroid cancer screening program was initiated in 2011 as a health policy after the nuclear accident. The risk of radiation-induced thyroid cancer was unlikely given the low radiation levels, but the thyroid cancer screening program has continued at 2-year intervals with a relatively high participation rate and is now in its fifth round. It is therefore crucial to clarify whether those targeted for screening understand the disadvantages of screening, and to identify factors that influenced their decision to participate.

**Methods:**

We conducted an anonymous mail-based questionnaire among young people from Fukushima Prefecture (subjects) and a neighboring prefecture that was not targeted for screening (non-subjects). We asked them about the significance of the thyroid cancer screening in Fukushima Prefecture, their reasons for accepting or refusing screening, their perception of the harms of screening, and their opinions on thyroid examination at school. We compared the results of the questionnaire between subjects and non-subjects and between examinees (who were screened) and non-examinees (who declined screening).

**Results:**

Only 16.5% of respondents were aware of the harms associated with thyroid cancer screening, with most perceiving that the benefits outweighed the harms. Comparison of subjects’ and non-subjects’ responses showed there were no significant differences between the two groups. Among subjects, there were also no differences in responses between examinees and non-examinees. The most common reason for participation in screening was that the screening was conducted in schools and perceived as obligatory.

**Conclusions:**

These results highlighted a serious ethical issue in that school-based screening leads to making young people think that it is mandatory screening in an opt-out and default setting manner, with a lack of knowledge about the disadvantages of screening. Based on the autonomy of the subjects and the ethical principle of the post-disaster, surveys after a nuclear disaster should be conducted in an opt-in style without an opt-out style such as school-based screening.

**Supplementary Information:**

The online version contains supplementary material available at 10.1186/s12885-022-09341-6.

## Background

Screening for various diseases, including cancer, has both benefits and harms, such as overdiagnosis. Provision of adequate information is therefore important in making decisions about whether to undergo screening. The harm associated with overdiagnosis is greater in children and adolescents than in adults, meaning that factors that aid decision-making about screening for children and adolescents require more consideration [1]. However, after a nuclear accident, residents may be screened for radiation-related cancers, especially radiation-induced thyroid cancer, as part of a health survey. Media and social networks may amplify residents’ radiation-related fears, and screening is often regarded as necessary for scientific and social reasons, without considering its possible harmful effects [2, 3].

More than 10 years have passed since the Fukushima Daiichi Nuclear Power Plant accident that occurred immediately after the Great East Japan Earthquake and tsunami disaster in March 2011. It was difficult to communicate sufficiently with residents about the health risks associated with radiation exposure for some time after the Fukushima nuclear accident [3, 4]. The level of radiation exposure among Fukushima residents was expected to be much lower than that among residents around Chernobyl after the 1986 accident [5]. However, based on experiences following the Chernobyl accident, residents in Fukushima were concerned about the increased risk for thyroid cancer among children and adolescents [6, 7]. Thyroid ultrasound examination was started in Fukushima Prefecture as part of the Fukushima Health Management Survey for all Fukushima residents (about 380,000 people) under 18 years old at the time of the accident [8]. This project started in October 2011, 6 months after the accident and was intended to monitor children’s health following the disaster. This timing meant that it was not possible to communicate with residents about the significance of the examination, including risks associated with radiation exposure in Fukushima, characteristics of juvenile thyroid cancer, and benefits and harms of thyroid cancer screening [7, 9]. The thyroid ultrasound examination in Fukushima Prefecture has been conducted as screening at 2-year intervals since 2011 [8], but was initially conceptualized as a support program for residents who were worried about the health effects of radiation exposure [7]. However, there was a lack of communication of key information before the examination, especially about overdiagnosis. The screening program used an active recruitment approach as an opt-out. All potential participants were notified about the venue and date of the examination, and examinations were also performed during school classes for school students aged 6 to 18 years old [10].

A global consensus against thyroid cancer screening emerged during the 10 years in which the thyroid ultrasound examination has been conducted in Fukushima Prefecture. For example, the rate of overdiagnosis following thyroid cancer screening in South Korea was described as “epidemic” and thyroid ultrasonography has been reported to increase the prevalence of thyroid cancer worldwide [11, 12]. Based on global reports, the US Preventive Services Task Force stated that thyroid cancer screening was not recommended for asymptomatic adults [13]. In 2018, the International Agency for Research on Cancer (IARC) stated that thyroid cancer screening was not recommended even after a nuclear accident [14]. A major reason for screening not being recommended is that evaluations suggested the harms of overdiagnosis by thyroid cancer screening outweighed the benefits. It has been reported that overdiagnosis can easily lead to overtreatment in the case of thyroid cancer [15]. When considering the harms of overdiagnosis, it is essential to keep the potential for overtreatment in mind. There are at least four categories of harms of overdiagnosis and overtreatment. The first is physical harm. Overdiagnosis is unnecessary, and both the detailed examination and the treatment, such as surgery or drug therapy, therefore become a physical burden. In addition, if complications or adverse effects occurring as a result of the examination or treatment are also harmful [16]. The second is psychological harm. Psychological burdens include anxiety about diagnosis and treatment and concerns about the prognosis and recurrence of the disease. The third is material harm, i.e., the cost of detailed examination and treatment and the time spent for examination and treatment [17]. The fourth is social harm. People diagnosed with cancer, even if overdiagnosis, may be discriminated in many areas, such as higher education, employment, love, marriage. There are also concerns about unfair deals in life insurance and loan contracts. These harms of overdiagnosis are likely to be more significant for younger people, as the rest of their lives will be longer [18, 19].

Four rounds of the thyroid ultrasound examination have been conducted in Fukushima Prefecture over the past 10 years, and a fifth round of opt-out screening is progressing despite these global trends. This examination has resulted in more than 200 thyroid cancer diagnoses (116 in the first round, 70 in the second, and 31 in the third) [20]. The observed age-specific prevalence of thyroid cancer was more than 30 times the expected rate in the first round, based on previous cancer registry data showing rates before screening [21]. The incidence of thyroid cancers detected by second-round screening was 48 cases per 105 person-years for the age group 18–20 years; this was nearly 50 times the incidence estimated for the same age group from the Japan cancer registry in 2001 to 2010 [22]. The United Nations Scientific Committee (UNSCEAR) concluded that the significant increase among screening participants relative to that expected was because of potential overdiagnosis and not the result of low-dose radiation exposure [5]. There is concern that this examination will continue to cause issues related to overdiagnosis. However, once initiated, a screening program is difficult to cancel because of conflicts of interest and economic policies, such as with the mass screening of newborns for neuroblastoma in Japan [23] and thyroid cancer screening in South Korea [11]. The thyroid cancer screening program in Fukushima Prefecture is also complicated because it started after the nuclear accident. As overdiagnosis is an emerging problem in recent health policy and practice [24], it is important to address its ethical implications. For example, it is necessary to clarify how well young people (as potential screening participants) understand the complex background and international changes in perspective about the harms of overdiagnosis following the thyroid ultrasound examination. The examination participation rate was high at 81.7% in the first round of screening, but showed a gradual decline to 71.0% in the second round and 61.3% in the third round [25]. However, a more detailed look at the participation rate showed the participation rate of school students remained close to 90%, even in the third round, but dropped sharply among those in the age group, those who had graduated from high school. This phenomenon may not occur if decisions about receiving screening are based on anxiety about radiation health risks. It is therefore essential to understand how residents perceived the health risks of radiation exposure and harms of thyroid cancer screening when they decided whether to undergo screening. This information is important in reconsidering the thyroid ultrasound examination program in Fukushima Prefecture as well as for future thyroid monitoring following a nuclear accident.

After a disaster, various research activities, including health surveys, tend to be conducted, but sometimes these activities are not beneficial for residents living in the affected areas [26, 27]. It is ethically important to determine why individuals make ongoing decisions to participate in potentially harmful examinations. To clarify factors that influenced the decision to undergo thyroid cancer screening in Fukushima Prefecture, we conducted a questionnaire survey among young people there, including those who received the thyroid examination and those who did not. We also sent the questionnaire to young people of the same generation in a neighboring prefecture who were not potential thyroid examination participants as a comparative control.

## Methods

We conducted an anonymous survey to clarify decision-making processes related to the thyroid ultrasound examination and associated ethical issues. The survey investigated three main areas.

1. Reasons why the examinee accepted the thyroid ultrasound examination.
2. Perceptions of the significance, benefits, and harms of the thyroid ultrasound examination.
3. The impact of the examination being conducted during school classes.

### Respondents

We sent an anonymous questionnaire to 2000 randomly selected young people aged 16–20 and 26–30 years as at January 1, 2020, who lived in Fukushima Prefecture and the neighboring Miyagi Prefecture. Those aged 21–25 years were excluded because they had not received screening at school during the previous 2 years and the participation rate is very low (around 10%-20%) among people over 21 years old. Most residents of these two prefectures were thought to have been affected by the Great East Japan Earthquake. The questionnaire was distributed via mail on January 7, 2020, and returned questionnaires with a postmark on or before February 15, 2020, were considered valid. Approval to conduct the survey was obtained from the Fukushima Medical University Ethics Committee (approval number: 2019–180). The questionnaire included a written explanation that returning a completed questionnaire would be considered provision of consent because the survey was completed anonymously and respondents were therefore not identifiable. We excluded from the analysis any respondents who provided data that differed from the data such as age in the resident registry and those who did not provide sufficient responses for analysis. Of the 601 young people (30.1%) who responded, 594 (29.7%) responses from those whose gender and age matched the population registry were considered valid. No difference in response rates between Fukushima and Miyagi prefectures (29.9% vs. 29.3%). This questionnaire survey was planned and conducted while the authors were affiliated with Fukushima Medical University.

### Questionnaire content

The questionnaire contained five categories of questions: 1) basic characteristics, 2) decision-making about participating in the thyroid ultrasound examination, 3) recognition of the benefits and harms of thyroid cancer screening, 4) perception of radiation-related health risks, and 5) the impact of conducting the examination during school classes.

The first category covered respondents’’ age, sex, and a question asking whether they were the subject of the examination (i.e. whether they were both resident in Fukushima Prefecture, and under 18 years old, at the time of the Fukushima accident).

The second category was only completed by those who were the subjects of the thyroid ultrasound examination in Fukushima Prefecture. This category contained four questions. i) Do you know the meaning of the thyroid ultrasound examination? If you answer yes, please describe it. ii) Did you have a thyroid ultrasound examination in the last 2 years? iii) Who made the decision whether you should have the examination? iv) Why did you have/not have the examination?

The third category asked all participants about: i) their knowledge of benefits and harms of the thyroid ultrasound examination, ii) magnitude of benefits and harms of the thyroid ultrasound examination using a five-point scale (more beneficial, beneficial, coequal, harmful, more harmful), and iii) knowledge about the IARC recommendation regarding thyroid cancer screening after a nuclear accident. For the analysis, “more beneficial” and “beneficial” were classified as “perceived beneficial” and “harmful” and “more harmful” were classified as “perceived harmful.”

For the fourth category, we measured participants’ perception of the potential health effects of radiation exposure based on their responses to two questions, with responses on a four-point scale from very unlikely to very likely [28, 29]. We investigated the possibility of delayed effects by asking, “What do you think is the likelihood of damage to your health (e.g., cancer onset) in later life as a result of your recent level of radiation exposure?” The second question concerned the possibility of genetic effects: “What do you think is the likelihood that the health of your future (i.e., as-yet unborn) children and grandchildren will be affected as a result of your recent level of radiation exposure?” For the analysis, “very unlikely” and “unlikely” were classified as lower risk perception and “likely” and “very likely” were classified as higher risk perception.

For the fifth category, we asked all participants to rate four statements about their perception of the impact of the examination conducted at school on a five-point scale from likely to unlikely. The statements were: i) Examination at school (during classes) makes you perceive it as a good thing; ii) Examination at school makes you believe it is somewhat mandatory; iii) Examination at school makes it difficult to refuse to have the examination; and iv) The presence of people who were not attending the school examination made you feel as if there was something wrong.

### Analysis

We used the chi-square test, Mann–Whitney U test, or Fisher's exact test to compare basic characteristics, knowledge of harms of the examination, and risk perception between the subject and the non-subject groups, and between those who received the examination (examinees) and those that did not (non-examinees). Reasons for receiving or not receiving the examination and the impact of school-based examination were explored using descriptive statistics.

## Results

Table 1 presents a summary of the results of the comparisons of knowledge and perception between the examinees and non-examinees and between the subjects and non-subjects. There was no difference in the male to female ratio between the examinees and non-examinees or between the subjects and non-subjects. However, the mean age of the non-subjects was higher than that of the subjects (*P* < 0.01), and the mean age of non-examinees was higher than that of examinees (*P* < 0.01). This was because subjects were aged 18 years or younger at the time of the nuclear accident. This meant that 75% of the population from Fukushima Prefecture aged 26–30 years who returned the questionnaire were non-subjects, whereas 96% of those aged 16–20 in Fukushima Prefecture were subjects. 95% of the population aged both 16–20 and 26–30 years in Miyagi Prefecture were non-subjects.

**Table 1:** The comparisons of knowledge and perception between the examinees and non-examinees and between the subjects and non-subjects

	Subjects			Non-subjects
	All^a^	Examinees	Non-examinees	
n	297	170	102	289
Age, mean, SD	19.2, 3.3**	18.0, 2.1*	21.9, 4.0*	25.6, 5.0**
Sex, male/female	142/155	79/91	50/52	132/157
Knowledge of the meaning of TUE (%), Yes, No	59.5, 40.5	65.5, 34.5	54.9, 45.1	-
Knowledge of existence of benefits and harms in TUE, n (%)	49 (16.5)	25 (14.9)	23 (23.0)	40 (13.8)
Knowledge of the IARC recommendation, n (%)	34 (11.4)	19 (11.2)	13 (12.7)	36 (12.5)
Estimation of benefits and harms in TUE				
Benefits are greater than harms, n (%)	132 (44.4)	79 (46.5)	42 (41.2)	123 (42.6)
Benefits are almost equal to harms, n (%)	83 (27.9)	50 (29.4)	27 (26.5)	64 (22.1)
Harms are greater than benefits, n (%)	13 (4.4)	7 (4.1)	5 (4.9)	14 (4.8)
Unknown, n (%)	66 (22.2)	33 (19.4)	26 (25.5)	86 (29.8)
Higher risk perception of delayed effect, n (%)	93 (31.3)**	56 (32.9)	35 (34.3)	138 (48.3)**
Higher risk perception of genetic effect, n (%)	63 (21.2)**	38 (22.4)	23 (22.5)	106 (36.8)**

^a^All subjects includes examinees, non-examinees, and unknown

*SD* Standard deviation, *TUE* Thyroid ultrasound examination, *IARC* International Agency for Research on Cancer

^*^P < 0.01 between examinees and non-examinees. **P < 0.01 between subjects and non-subjects

Overall, 40.5% of respondents who were subjects of the thyroid ultrasound examination did not know the purpose of the examination. There was no significant difference in knowledge between examinees and non-examinees. Furthermore, about half of those who said they knew the purpose of the examination provided incorrect descriptions; for example, “This examination measures the radiation level of the thyroid gland” (data not shown).

Regarding decision-making about having the examination, 54.2% of examinees indicated the decision was made by their parents, and 15.3% made the decision themselves. In contrast, 57.0% of non-examinees made the decision themselves, and 9.0% reported their parents made the decision. We believed that this corresponded to the decline in the examination participation rate after graduating from high school. Common reasons for having the examination (multiple-choice question) were: 1) this was an examination done at school (29.6%), 2) concern about radiation effects (13.5%), and 3) wanting to be relieved by undergoing the examination (11.1%). Common reasons for not having the examination were: 1) time-consuming (11.1%) and 2) no worry about radiation exposure (11.1%). However, no participant indicated “harms of the examination” was a reason for not receiving the examination.

Table 1 also shows that only 16.5% of respondents in the examinee and non-examinee groups knew about the harms of thyroid ultrasound examination. The proportion of those who knew about the harms was slightly smaller in the examinee group, but the difference between the groups was not significant. In addition, few people knew about the IARC recommendation against thyroid cancer screening, and there was no significant difference between groups. Furthermore, both examinees and non-examinees showed a tendency to overestimate the benefits compared with the harms of screening. The results regarding knowledge about the benefits and harms of the thyroid examination were similar for the non-subjects.

There was no significant difference between examinees and non-examinees in responses to questions about the delayed and genetic effects caused by radiation exposure. However, the perceptions of risk for both delayed and genetic effects were significantly higher in non-subjects than in subjects (% of higher delayed risk perception: 31.3% vs. 48.3% (*P* < 0.01); % of higher genetic risk perception: 21.2% vs. 36.8% (*P* < 0.01)).

**Fig. 1:**
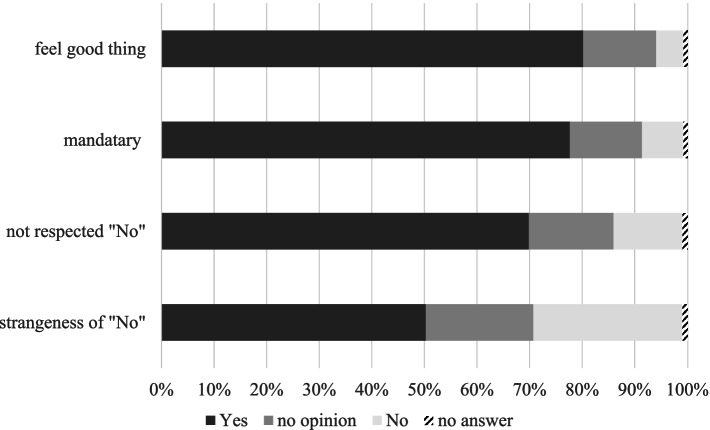
Impact of the school-based thyroid examination. The percentage of “Yes” responses are shown for the following items related to the recognition of a school-based examination. i) 80% of participants perceived the examination was a good thing. ii) 78% of participants considered the school-based examination was somewhat mandatary. iii) 70% of participants said that their preference not to have the examination was not respected. iv) About half of the participants felt there was something wrong with those who did not have the examination. The remaining percentages for each item show responses of “no”, “no opinion”, or “no answer”

Figure 1 shows the results of the analysis of items covering the impact of a school-based examination. In summary, these were:

i) About 80% of participants perceived the examination was good because it was a school-based examination.
ii) Around 78% of participants considered the school-based examination was somewhat mandatary.
iii) Overall, 70% of participants said that their preference not to have the examination was not respected.
iv) Finally, about half of the participants said they perceived something wrong about those who did not have the examination.

There were no differences in these opinions between the subject and non-subject groups, or between the examinee and non-examinee groups.

## Discussion

The risks for cancer due to radiation depends on the radiation dose, various activities to measure the radiation dose were conducted to respond to residents’ health concerns after the Fukushima nuclear accident [30]. The radiation doses were expected to be extremely low in Fukushima [5], meaning that implementation of thyroid cancer screening did not logically correspond to the concerns about the health effects of radiation exposure. However, it has been noted that the high participation rate in the thyroid ultrasound examination in Fukushima Prefecture may reflect concerns about the health effects of radiation exposure among the parents of examination participants, especially mothers [7, 9]. In contrast, this study directly asked young people (who were the subjects) about the most common reason for having the examination, which was because it was performed at school. This does not support the suggestion that many people had the examination to relieve anxiety about the health risk of thyroid cancer due to radiation exposure. This was also supported by our result that there was no significant difference in perception of risk about radiation health effects (especially the risk for developing cancer such as delayed effects) between examinees and non-examinees. Furthermore, among those that had the examination, slightly more than half reported their parents made the decision about participation. However, among those that did not have the examination, slightly more than half made this decision themselves. The thyroid examination in Fukushima Prefecture requires a parent’s signature for those under 15, but those aged 16 and older can sign for themselves. However, for 16- and 17-year-olds, a signature from both the young adult and a parent is preferred. Therefore, parents’ perception may influence their decision to have the examination as well as the impact of the examination being conducted at school.

To support proper decision-making as to whether young people should have the screening examination, it is essential that they understand the significance, benefits, and harms of the examination. Our results showed that 40.5% of the thyroid ultrasound examination subjects did not know the purpose of the examination, and there was no significant difference in this knowledge between examinees and non-examinees. In addition, the free-form answers showed that 47.8% of respondents who had this knowledge misunderstood the meaning of the examination (e.g., “This examination measures the radiation level of the thyroid gland”). This highlighted that the subjects, even examinees, of the thyroid ultrasound examination often did not correctly understand the purpose of the examination.

Most subjects were also unaware of the harms of thyroid cancer screening, and the percentage of those who knew there were harms associated with thyroid cancer screening was the same for examinees and non-examinees. Both examinees and non-examinees considered the benefits were greater than the harms. However, no respondents cited these harms as a reason for not having the examination. These results suggested that potential thyroid examinees are somehow encouraged to undergo the examination in the decision-making process. Recently, it has been reported that examinees underestimate the disadvantages and overestimate the benefits of screening, testing, and intervention [31, 32]. It has also been noted that recognition of harms is important in decision making about breast cancer screening [33, 34]. A qualitative study involving Korean women reported that many of them were unaware of the potential harm of overdiagnosis associated with thyroid ultrasound screening [35].

We found that the risk perceptions of health effects due to radiation exposure were significantly higher in non-subjects than in subjects. This suggested that non-subjects (who were relatively far from the nuclear accident site) overestimated the radiation health risk compared with subjects. Similar results were shown in a survey by the National Institute of Advanced Industrial Science and Technology, where the risk perception of the general population in Tokyo was significantly higher than that of residents in the evacuation area in Fukushima Prefecture [36]. These results indicated that the subjects had opportunities to access information and education on radiation health risks. In contrast, there was a non-significant difference in the awareness of the existence of harms of thyroid cancer screening between the subjects and non-subjects. Both groups also overestimated the benefits compared with the harms of thyroid cancer screening. The subjects had received an explanation letter with guidance about taking the thyroid examination, whereas non-subjects did not receive a guidance letter. However, the benefit-harm perception did not differ between the two groups. This suggested that the explanation and communication through the explanation letter before decision-making about having the examination was insufficient.

This study showed that the examination at school led to the perception that the examination was a good thing or mandatory, and that preferences to not have the examination were not respected. This perception was not specific to the subjects of the thyroid ultrasound examination, but extended to non-subjects, which suggests that it is a common tendency among Japanese young people. Our data revealed that the methodology whereby the examination was conducted at school during classes could influence decision-making about having the examination. This was supported by the sharp decline in the thyroid ultrasound examination participation rate after graduating from school [25]. These results indicated that the school-based examination became a “default,” as an opt-out method was used. The impact of opt-in and opt-out methods on decision making has been reported in the issue of organ donation [37, 38]. Opt-out in organ donation is a recruitment approach that presumes all individuals are willing to donate organs after death unless they specifically "opt-out" of doing so. This is also known as "presumed consent." Opt-in is the opposite of opt-out because no one is presumed to be a willing donor unless they have expressed this wish. This is also known as an "express consent". The organ donation rate is high in countries with opt-out policies and low in opt-in countries.

Our results indicate that the thyroid examination in Fukushima Prefecture has set the default as participation even though with a written explanation of screening harms and signed consent because the examinations are held at school for 6–18 years old. To solve this ethical challenge, it would be helpful if school-based screening stopped so that the subjects can understand the harm of overdiagnosis. This will allow them to think autonomously about whether to have the examination, including when they go to public facilities based on opt-in approach for screening. In addition to education on radiation health risks, a health literacy education on the merits and demerits of medical examinations in health management, especially on the risks of overdiagnosis, is essential given the potential for future nuclear disasters. Even with major benefits of organ transplants, perspectives of the methodology of choosing opt-out or opt-in are divided. From an ethical perspective, care must be taken in adopting the opt-out method if a program has some potential harms. In organ transplantation, the ethical validity of changing from an opt-in to opt-out format is currently being discussed [39]. Although there may be ethical issues associated with an opposite change, thyroid cancer screening should change from an opt-out to opt-in to resolve the ethical challenges associated major harms such as overdiagnosis.

This study had several limitations. First, we identified the subjects/non-subjects and examinees/non-examinees based on responses to category 1. Based on the respondents’ age and address, most of these responses were thought to be correct. However, some respondents might have mistakenly answered about another examination rather than the thyroid examination. Further, it is not possible to confirm by the Fukushima Health Management database whether or not the respondents have had the examination. Some of non-subjects may also have the opportunity to know the thyroid examination without the screening project. Further, non-examinees might have had a thyroid examination more than two years previously, which could have biased the results. Second, the response rate in this study was relatively low (30%), the representativeness of the target populations was therefore uncertain. However, the majority of respondents were unaware of the disadvantages of thyroid examination as a cancer screening tool, a finding that is likely to be unaffected by response rate bias. Third, the age of non-subjects was higher than that of the subjects, and perception of the risk of health effects due to radiation exposure might have been influenced by age. However, there has been no reports of any large differences in radiation health risk perception between the ages of 16–20 and 26–30 years. Fourth, this study could not obtain information directly from the respondents' parents. The study results therefore may not purely reflect the perception of young people but also those of their parents.

## Conclusions

**Fig. 2:**
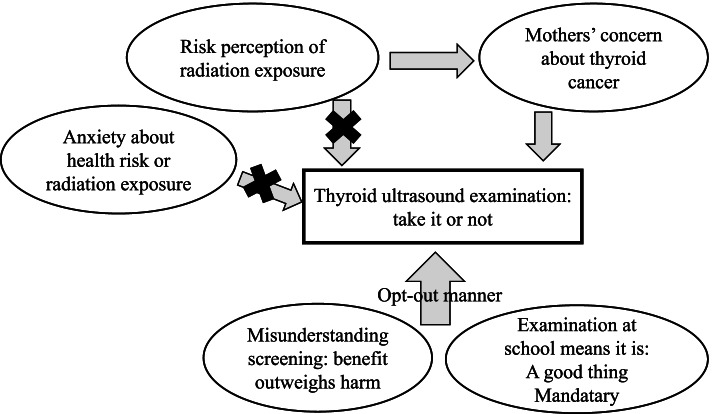
Decision-making process and potential influencing factors for young subjects of the thyroid ultrasound examination in Fukushima Prefecture. There were several potential factors that influenced whether the subjects took the thyroid ultrasound examination after the nuclear accident. The "✖" indicates factors that were not found to be affected by this study

We summarized the decision-making process and potential influencing factors for young subjects of the thyroid ultrasound examination in Fukushima Prefecture in Fig. 2. After a nuclear accident, residents are naturally concerned about the health effects of radiation exposure and tend to perceive high risks of radiation-related cancer. However, our results indicated there were no relationships in these factors. Screening as a health survey tends to be conducted as a policy to resolve these concerns, and opt-out style screening may therefore result in the misunderstanding that screening benefits outweigh the harms. This is especially true for screening based in schools. This is not limited to thyroid cancer screening, because screening to detect any disease may not be beneficial for residents following a disaster [10, 26]. To properly respond to anxieties of affected residents, it is essential to communicate key aspects to residents, including: 1) purpose of the examination, 2) balance between the benefits and harms of the examination, 3) characteristics of the target disease, and 4) voluntary participation. In addition, based on the principle of the post-disaster code of conduct, investigations and surveys after a disaster should be conducted in an opt-in style.

## Supplementary Information

**Additional file 1. **

## Data Availability

Data other than those shown in tables, figures, and supplementary files are not publicly available.
