# Supplementary material for: Young people’s perspectives of thyroid cancer screening and its harms after the nuclear accident in Fukushima Prefecture: a questionnaire survey indicating opt-out screening strategy of the thyroid examination as an ethical issue
Source: BMC Cancer. 2022 Mar 3;22:235. doi: 10.1186/s12885-022-09341-6 (PMC8896110; doi:10.1186/s12885-022-09341-6)
Supplement: Supplementary file 1 — Additional file 1. [file 12885_2022_9341_MOESM1_ESM.docx]

Questionnaire

1) Age ( )

2) Gender

1 □ man

2 □ woman

3) Are you a subject of the thyroid examination in the Fukushima Health Management Survey? (The subjects of the thyroid examination lived in Fukushima prefecture at the time of the Great East Japan Earthquake and were 18 and under years old.)

1 □ Yes (⇒ Please go to 4)

2 □ No (⇒ Please go to 10)

4) Do you know the meaning of the thyroid examination? If you know, please write down the reason.

1 □ I know (Reason: )

2 □ I don't know

5) Did you have the thyroid examination in the last two years?

1 □ Yes (⇒ Please go to 6)

2 □ No (⇒ Please go to 8)

3 □ I don't know (⇒ proceed to 10)

6) At that time, who made the decision you should have the thyroid examination?

1 □ Parents decided (⇒ Please go to 10)

2 □ I decided (⇒ Please go to 7)

3 □ I consulted to parents and decided (⇒ Please go to 7)

4 □ I don't know (⇒ Please go to 10)

7) What is the reason for your participation in the thyroid examination? Would you please select all that apply?

1 □ Because it is done at school

2 □ Because my friends around me are also taking

3 □ Because it was recommended by the people around you (family, friends, familiar

specialists, etc.)

4 □ I'm worried about the health effects of radiation

5 □ I want to be relieved to undergo the thyroid examination

6 □ I want to know if I have a disease such as thyroid cancer

7 □ I will take the thyroid examination without thinking well when I receive

the notification letter.

8 □ I think I have to undergo the thyroid examination (obligation)

9 □ I want to cooperate with the thyroid examination

10□ Others( )

8) [Please answer only for those who answered "I did not have the thyroid examination" in 5)] Who made the decision you should not have the thyroid examination?

1 □ Parents decided (⇒ Please go to 10)

2 □ I decided (⇒ Please go to 9)

3 □ I consulted my parents and decided (⇒ Please go to 9)

4 □ I don't know (⇒ Please go to 10)

9) What is the reason for not taking the thyroid examination? Would you please select all that apply?

1 □ Because it takes time

2 □ Because my friends around me haven't taken

3 □ Because it was recommended by the people around you (family, friends, familiar

specialists, etc.)

4 □ I don't understand the significance of the thyroid examination

5 □ I'm not particularly worried

6 □ I thought I didn't have to take the thyroid examination

7 □ I think there are some disadvantages of the thyroid examination

8 □ Other( )

10) The thyroid examination performed in Fukushima Prefecture is to observe the thyroid gland with an ultrasonic examination device and screen nodules containing cancer in the thyroid gland. There are pros and cons to screening for all illnesses, not just this one. Did you know that this thyroid examination also has its advantages and disadvantages?

1 □ I didn't know

2 □ I knew

11) Which do you feel is the magnitude of benefits and harms of the thyroid examination performed in Fukushima Prefecture? Would you please select the number that applies?

| More beneficial | Beneficial | Coequal | Harmful | More harmful | I do not know |
| --- | --- | --- | --- | --- | --- |
| 1 | 2 | 3 | 4 | 5 | 6 |

12) The IARC (International Agency for Research on Cancer) makes the following two recommendations regarding thyroid examinations after a nuclear accident, considering the advantages and disadvantages, as a reference for future nuclear accidents.

One is that it is not recommended to perform a mass thyroid examination after a nuclear accident. Second, when radiation exposure is high, IARC recommend that you consider consulting with each individual to see if they should have a thyroid examination. In the accident in Fukushima, the radiation exposure level, including people who lived in the evacuation order area, is estimated to be lower than the value indicated in the second recommendation.

Did you know the content of this IARC proposal?

1 □ I didn't know

2 □ I’ve heard of it

3 □ I knew

13) At elementary, junior high, and high schools in Fukushima Prefecture, the thyroid examination has been conducted at school during class hours. What are your thoughts on this? Please select the number that applies.

|  | Very likely | Likely | Neither | Unlikely | Very unlikely |
| --- | --- | --- | --- | --- | --- |
| 1．Examination at school (during classes) makes you perceive it as a good thing. | 1 | 2 | 3 | 4 | 5 |
| 2．Examination at school (during classes) makes you believe it is somewhat mandatory. | 1 | 2 | 3 | 4 | 5 |
| 3．Examination at school (during classes) makes it difficult to refuse having the examination. | 1 | 2 | 3 | 4 | 5 |
| 4．The presence of people who are not attending the school examination made you feel as if there was something wrong. | 1 | 2 | 3 | 4 | 5 |
